# An angel or a devil? Current view on the role of CD8^+^ T cells in the pathogenesis of myasthenia gravis

**DOI:** 10.1186/s12967-024-04965-7

**Published:** 2024-02-20

**Authors:** Yong Peng, Huan Yang, Quan Chen, Hong Jin, Ya-hui Xue, Miao-qiao Du, Shu Liu, Shun-yu Yao

**Affiliations:** 1 Department of Neurology, Affiliated First Hospital of Hunan Traditional Chinese Medical College, Zhuzhou, 412000 Hunan China; 2 Department of Neurology, The Third Affiliated Hospital of Hunan University of Chinese Medicine, Zhuzhou, 412000 Hunan China; 3 Department of Neurology, Xiangya Hospital, Central South University, Changsha, 410008 Hunan China

**Keywords:** Myasthenia gravis, Experimental autoimmune myasthenia gravis, CD8^+^ T cells, Effector T cells, Regulatory T cells

## Abstract

**Background:**

Myasthenia gravis (MG) and the experimental autoimmune MG (EAMG) animal model are characterized by T-cell-induced and B-cell-dominated autoimmune diseases that affect the neuromuscular junction. Several subtypes of CD4^+^ T cells, including T helper (Th) 17 cells, follicular Th cells, and regulatory T cells (Tregs), contribute to the pathogenesis of MG. However, increasing evidence suggests that CD8^+^ T cells also play a critical role in the pathogenesis and treatment of MG.

**Main body:**

Herein, we review the literature on CD8^+^ T cells in MG, focusing on their potential effector and regulatory roles, as well as on relevant evidence (peripheral, in situ, cerebrospinal fluid, and under different treatments), T-cell receptor usage, cytokine and chemokine expression, cell marker expression, and Treg, Tc17, CD3^+^CD8^+^CD20^+^ T, and CXCR5^+^ CD8^+^ T cells.

**Conclusions:**

Further studies on CD8^+^ T cells in MG are necessary to determine, among others, the real pattern of the Vβ gene usage of autoantigen-specific CD8^+^ cells in patients with MG, real images of the physiology and function of autoantigen-specific CD8^+^ cells from MG/EAMG, and the subset of autoantigen-specific CD8^+^ cells (Tc1, Tc17, and IL-17^+^IFN-γ^+^CD8^+^ T cells). There are many reports of CD20-expressing T (or CD20 + T) and CXCR5^+^ CD8 T cells on autoimmune diseases, especially on multiple sclerosis and rheumatoid arthritis. Unfortunately, up to now, there has been no report on these T cells on MG, which might be a good direction for future studies.

## Introduction

Myasthenia gravis (MG) and its animal model, experimental autoimmune MG (EAMG), are T-cell-driven, autoantibody (Ab)-mediated disorders affecting transmission in neuromuscular junctions [1–5]. As the treatment of MG has remained a burden for patients, finding better treatments is necessary [6]. Abs in patients with MG are against the muscle nicotinic acetylcholine receptor (AChR), muscle-specific tyrosine kinase (MuSK), and lipoprotein-related protein 4 [1, 3, 5, 7], and are secreted by B cells and plasma cells with assistance from CD4^+^ T cells [1, 8], resulting in complement anti-AChR antibodies damaging the neuromuscular junctions and inducing MG symptoms such as ptosis, dysphagia, limb weakness, and even dyspnea [4, 9].

A possible mechanism of MG might be that these autoreactive Abs bind to functional molecules in the postsynaptic membrane, impairing signal transmission in these synapses [10]. Currently, a few new Abs have been confirmed to affect patients with MG in several clinical trials. Regarding existing treatments, eculizumab has been approved in many countries [11–13], zilucoplan has shown better results than eculizumab in a phase II clinical trial [14], and efgartigimod has been successful in a phase III clinical trial [15]. Moreover, immunomodulatory drugs, such as terifunomide [16, 17], have also shown benefits for MG treatment in EAMG [16].

Many subtypes of CD4^+^ T cells contribute to the pathogenesis of MG, including T helper 1 (Th1), Th17, Th22, follicular Th (Tfh), Th_CD103_, and regulatory T (Treg) cells; however, previous reports focused on CD4^+^ T cells [18–30]. Major histocompatibility complex (MHC) class I-restricted CD8^+^ T cells act as both effectors and/or regulators in various autoimmune diseases, such as multiple sclerosis (MS) and its animal model, experimental autoimmune encephalomyelitis (EAE) [31–33], and animal models of uveitis—experimental autoimmune uveitis (EAU) [34]. However, in Theiler’s murine encephalomyelitis virus-induced demyelination, CD8^+^ T cells played a regulatory role predominantly*.* MHC class I and CD8^+^ T cell deficiency in β2-m^−/−^ mice inhibited clinical scores in an animal model of type I diabetes and Ab-mediated autoimmune disease, such as systemic lupus erythematous disease.

Indeed, increasing evidence supports that CD8^+^ T cells also play a critical role in the pathogenesis and treatment of MG [13, 35, 36] and EAMG [37], even though this evidence is fragmented and unsystematic [38]. Herein, we investigate CD8^+^ T cells in MG, focusing on their potential effector and regulatory roles, as well as on evidence (peripheral, in situ, cerebrospinal fluid (CSF), and under different treatments), T-cell receptors (TCR) usage, cytokine and chemokine expression, cell marker expression, regulatory T cells, and Tc17 cells.

## Evidence of CD8^+^ T cells in patients with MG

### Peripheral CD8^+^ T cells in patients with MG with and without treatment

The first report on peripheral CD8^+^ T cells in patients with MG was published in 1988, although there were no reported significant differences in peripheral blood lymphocyte subsets (including CD4^+^, CD8^+^, and the ration of CD4^+^/CD8^+^) in patients with or without a thymus [39].

Activated CD8^+^ and CD4^+^ T cells co-exist in the peripheral blood of patients with MG, and the numbers of CD8^+^ and CD4^+^ T cells are related to the severity of the disease [40]. Furthermore, in both patients with MG and healthy controls, CD8^+^ peripheral blood mononuclear cells (PBMCs) and CD8^+^ lines responded vigorously to autologous antigen (Ag) (including AchR), which can activate CD4^+^ cells. The CD8^+^ cell lines responded equally well to the different Ag- and phytohemagglutinin-activated CD4^+^ cells [41]. Moreover, one report showed that the amount or function of CD8^+^ T cells was destroyed in patients with MG. The authors also confirmed that the inhibitory function of CD8^+^ cells and exogenous interleukin (IL)-2 completely restored this suppression in vitro. The expression of CD8^+^ was decreased in 71% of patients with MG [42]. However, another report showed that the percentages of CD8^+^ T cells in peripheral blood were not significantly different among three groups: ocular MG (OMG), generalized MG, and healthy controls [43].

MG usually coexists with several diseases. In autoimmune thyroid diseases, compared with thyroid antibody (TAb)-negative patients, TAb-positive patients appeared to have a higher prevalence of OMG, higher percentages of CD8^+^ CD28^+^ cells, lower AChR-Ab titers, and percentages and absolute counts of total CD8^+^ T cells [44]. In patients with Sjogren’s syndrome, CD8^+^ cells and their two subsets significantly increased in untreated patients with MG. In a female patient with MG and Good’s syndrome that presented with paroxysmal nocturnal hemoglobinuria, CD8^+^ T cells are directly responsible for inhibiting the growth of control PBMC. CD8^+^ T cells from patients with MG showed a reduced number of Vβ-TCR families (Vβ2, Vβ5.3, Vβ14, and Vβ20). CD8^+^ T cells are associated with damaging hematopoietic precursors as confirmed by co-culture experiments and spectratyping analyses [45].

In patients with MG, inflammatory cell infiltration was observed in the heart and skeletal muscles; however, the severity varied. The heart showed widespread inflammatory infiltrates containing multinucleated giant cells with massive myocardial degeneration. CD8^+^ cells were observed in both necrotic and non-necrotic muscle fibers. A limited number of mononuclear cells infiltrating the perimysial or perivascular regions were CD8^+^ cells. Muscle fibers, including normal-appearing ones, aberrantly express diffuse MHC class I on surface membranes (Table 1) [46].

**Table 1:** peripheral CD8 + T cells in patients of MG with and without treatment

Study ID	Sample	Treatment	CD8 percentage	CD4/CD8 ratio	CD8 subset	Significant findings of CD8 in MG
Machi 1988	MG	Total	39.9 ± 8.0	1.34 ± 0.43	N/A	None
	MG	Non-Tx	39.6 ± 7.4	1.41 ± 0.42	N/A	None
	MG	Tx	40.2 ± 9.3	1.24 ± 0.45	N/A	None
	HC	None	39.2 ± 5.5	1.40 ± 0.34	N/A	N/A
Kawanami 1990	MG	Total	N/A	N/A	N/A	N/A
	MG	None	29.7 ± 3.0	1.2 ± 0.16	N/A	N/A
	MG	Tx(− )PSL( +)	26.5 ± 2.1	1.3 ± 0.18	N/A	N/A
	MG	Tx( +) PSL( − )	25.8 ± 3.5	1.7 ± 0.31	N/A	N/A
	MG	Tx( +) PSL( +)	29.6 ± 1.1	0.94 ± 0.24	N/A	N/A
	HC Female	None	26.8 ± 1.7	1.5 ± 0.1	N/A	N/A
	HC male	None	28.4 ± 1.4	1.3 ± 0.1	N/A	N/A
Shimizu 1990	MG	Total	28.0 ± 10.5	1.8 ± 1.0	CD8 + CD11 + , CD8 + CD11−	CD8 + CD11 + , CD8 + CD11−
	MG	Tx( +) PSL( − )	22.8 ± 4.3	2.2 ± 0.8	CD8 + CD11 + , CD8 + CD11−	CD8 + CD11 + , CD8 + CD11-
	MG	Tx( − )PSL( +)	30.8 ± 12.6	1.5 ± 1.0	CD8 + CD11 + , CD8 + CD11−	CD8 + CD11 + , CD8 + CD11-
	MG	Tx( +) PSL( − )	25.2 ± 6.5	2.1 ± 0.9	CD8 + CD11 + , CD8 + CD11−	CD8 + CD11 + , CD8 + CD11-
	MG	Tx( +) PSL( +)	30.2 ± 12.1	1.7 ± 1.2	CD8 + CD11 + , CD8 + CD11−	CD8 + CD11 + , CD8 + CD11−
Fujii 1991	MG group A	Tx( +) PSL( +)	39.3 ± 9.2	1.23 ± 0.36	N/A	N/A
	MG group B	Tx( +) PSL( − )	33.3 ± 13.6	1.89 ± 0.70	N/A	N/A
	HC	None	39.2 ± 5.5	1.40 ± 0.34	N/A	N/A
Tanaka 2009	MG	Tx( +)	Mean 21	N/A	N/A	N/A
	MG	None	Mean 14	N/A	N/A	N/A

There have been several reports on the cell culture of CD8^+^ T cells in patients with MG. Out of all T-cell lines tested, CD4^+^ cells were only present in seven AChR-specific T-cell lines obtained from three patients with MG. In addition, predominant CD4^+^CD8^—^ (15/38) and a few CD4^−^CD8^+^ cells (5/38) were observed in 38 AchR or recombinant mammalian AchR α chain peptide (X4) T-cell lines obtained from 11 healthy individuals. Similarly, out of seven human AchR β subunit-specific T-cell lines obtained from patients with MG (four lines) and healthy controls (three lines), only one CD4^+^ and CD8^+^ (ratio around 1:1) were observed.

### CD8^+^ T cells in patients with MG under different treatments

Thymoma is a non-malignant thymus tumor characterized by many non-malignant lymphocytes usually associated with MG. The percentage of mature single-positive T lymphocytes (including CD4^−^CD8^+^ cells) in thymomas with MG increased significantly compared with that without MG. In addition, compared with the proportion of CD8^+^ cells in thymocytes of patients with MG without thymoma, those inpatients with MG with thymoma increased significantly during the incubation period with IL-2.

Thymus responses to T-cell differentiation and maturation. Thymectomy (Tx) improved the clinical outcomes in patients with MG with or without thymomas [47, 48]. After Tx and steroid therapy, CD8^+^ T cells decreased immediately after Tx and returned to pre-treatment levels within three weeks. These results are similar to those of other studies [49–51]. Subsequently, the percentage of CD8^+^CD11^−^ cells decreased, whereas that of CD8^+^ CD11^+^ cells increased after Tx and prednisolone treatment. The absolute number of CD45RA^+^CD8^+^ CD62L^+^ and CD45RO^+^CD8^+^T cells decreased significantly in the blood of patients with MG who underwent Tx [49]. CD8^+^ T cells in patients with MG increased after Tx, whereas a decrease was observed in prealbumin and albumin levels [52]. Naïve cytotoxic T cells (CD8^+^CD27^+^CD28^+^) were reduced in patients who underwent thymectomy [51]. CD8^+^ T-cell counts were markedly higher in the conventional trans-sternal extended Tx (TS) group on postoperative days 1 and 3 but remained relatively stable in the video-assisted thoracoscopic surgery group. On postoperative day 7, CD4^+^ counts were similar in the TS and video-assisted thoracoscopic surgery groups, whereas CD8^+^ counts remained higher in the TS group [53].

### CD8^+^ T cells in patients with MG with and without thymoma

Immunosuppressive therapy, including steroids and tacrolimus, is a common treatment for MG. The percentage of DR^+^ and CD8^+^/DR^+^ T cells in the blood increased and the level of CD4^+^ T cells decreased when patients with MG received immunosuppressive therapy (steroids alone or in combination with azathioprine). Tacrolimus (FK506) is an immunosuppressive agent similar to cyclosporin A that inhibits the action of calcineurin, a serine/threonine phosphatase, thereby suppressing IL-2 production. Tacrolimus treatment significantly attenuated T-cell receptor excision circle (TREC) levels in cultured CD4^–^CD8^+^ cells, but the total cell counts did not change significantly. In addition, compared with that in patients with MG without thymoma, levels of CD8^+^ T cells in patients with MG with thymoma decreased significantly after tacrolimus therapy [54]. Additionally, P-glycoprotein (P-gp) actively transports glucocorticoids (GC) out of target cells, thereby reducing its efficacy. Compared with patients with MG without GC therapy, P-gp function in CD8^+^ T cells was higher in patients with MG with long-term GC therapy (Table 2) [50].

**Table 2:** peripheral CD8 + T cells in patients of EAMG with and without treatment

Study ID	Sample	Treatment	CD8 percentage	CD4/CD8 ratio	CD8 subset	Significant findings of CD8 in MG
Fujii 1988	Lewis rats	Total	39.9 ± 8.0	1.34 ± 0.43	N/A	None
	MG	Non-Tx	39.6 ± 7.4	1.41 ± 0.42	N/A	none
	MG	Tx	40.2 ± 9.3	1.24 ± 0.45	N/A	none
	HC	None	39.2 ± 5.5	1.40 ± 0.34	N/A	N/A
Kawanami 1990	MG	Total	N/A	N/A	N/A	N/A
	MG	None	29.7 ± 3.0	1.2 ± 0.16	N/A	N/A
	MG	Tx(−)PSL( +)	26.5 ± 2.1	1.3 ± 0.18	N/A	N/A
	MG	Tx( +) PSL(−)	25.8 ± 3.5	1.7 ± 0.31	N/A	N/A
	MG	Tx( +) PSL( +)	29.6 ± 1.1	0.94 ± 0.24	N/A	N/A
	HC Female	None	26.8 ± 1.7	1.5 ± 0.1	N/A	N/A
	HC male	None	28.4 ± 1.4	1.3 ± 0.1	N/A	N/A
Shimizu 1990	MG	Total	28.0 ± 10.5	1.8 ± 1.0	CD8^+^CD11^+^, CD8^+^CD11^−^	CD8^+^CD11^+^, CD8^+^CD11^−^
	MG	Tx( +) PSL(−)	22.8 ± 4.3	2.2 ± 0.8	CD8^+^CD11^+^, CD8^+^CD11^−^	CD8^+^CD11^+^, CD8^+^CD11^−^
	MG	Tx(−)PSL( +)	30.8 ± 12.6	1.5 ± 1.0	CD8^+^CD11^+^, CD8^+^CD11^−^	CD8^+^CD11^+^, CD8^+^CD11^−^
	MG	Tx( +) PSL(−)	25.2 ± 6.5	2.1 ± 0.9	CD8^+^CD11^+^, CD8^+^CD11^−^	CD8^+^CD11^+^, CD8^+^CD11^−^
	MG	Tx( +) PSL( +)	30.2 ± 12.1	1.7 ± 1.2	CD8^+^CD11^+^, CD8^+^CD11^−^	CD8^+^CD11^+^, CD8^+^CD11^−^
Fujii 1991	MG group A	Tx( +) PSL( +)	39.3 ± 9.2	1.23 ± 0.36	N/A	N/A
	MG group B	Tx( +) PSL(−)	33.3 ± 13.6	1.89 ± 0.70	N/A	N/A
	HC	None	39.2 ± 5.5	1.40 ± 0.34	N/A	N/A
Tanaka 2009	MG	Tx( +)	Mean 21	N/A	N/A	N/A
	MG	None	Mean 14	N/A	N/A	N/A

In summary, these studies provide strong evidence of the critical role of CD8^+^ T cells in the pathogenesis of patients with MG with or without treatment, including patients with MG with or without other diseases, with in situ CD8^+^ T cells in the heart and skeletal muscle and CD8^+^ T cells in the CSF, as well as those undergoing different treatments, such as Tx and immunosuppressive therapy. Although CD8^+^ T cells are important in the pathogenesis of MG, existing studies on CD8^+^ T cells are fewer than those for CD4^+^ T cells in relation to MG. We have previously reported that the classical T-cell culture system was favorable for CD4^+^ T cells rather than CD8^+^ T cells in EAE and EAU. Possibly, the major subset of CD8^+^ T cells is cytotoxic T-lymphocytes (CTL), which easily causes cell damage, even to themselves, and the expansion of CD8^+^ T cells in vitro requires support from CD4^+^ T cells and their secreted cytokines [31, 32, 34, 55]. Therefore, there is a similar condition in MG/EAMG, which is also a T cell-mediated autoimmune disease.

## Evidence of CD8^+^ T cells in EAMG

### Peripheral CD8^+^ T cells in patients of MG with and without treatment

The first report on peripheral CD8 + T cells in EAMG was also published in 1988, although there were no reported significant differences in peripheral blood lymphocyte subsets (including CD4 + , CD8 + , and the ration of CD4 + /CD8 +) in MG with or without a thymus. Ten acetylcholine receptor (AChR)-specific T cell clones from Lewis rats were studied. These clones had various AChR subunit and peptide specificities and proliferated in response to the antigen on the appropriate APC. All T cell clones were CD4 + CD8- and OX22-, helped anti-AChR antibody production by AChR-primed lymph node B cells, and could secrete IL-2. However, several lines of evidence suggest that IL-2 was not the lymphokine that mediated T cell help. B cells primed with native AChR and exposed in culture to very low concentrations of native AChR effectively presented the Ag to T cell lines, presumably owing to an uptake via Ag receptors; however, primed B cells were no more effective than non-specific APC at presenting a synthetic AChR peptide that is recognized by AChR-specific T cells but not by AChR-specific B cells. Increasing AChR doses to a certain level produced an antibody production response that was bell shaped and only stimulated by low AChR concentrations; higher AChR concentrations suppressed the antibody production response. The evidence suggested that AChR exerted its inhibitory effect through T cells, but not via IL-2.

An EAMG model is a valuable tool for studying the pathogenesis of MG. There is evidence in rat and mouse models to support the role of CD8^+^ T cells in the pathogenesis of MG. Ten AchR-specific T-cell clones from Lewis rats in an EAMG model were CD4^+^CD8^−^ and OX22 − . The EAMG model was generated by peritoneally injecting thymocytes from patients with MG into severe combined immunodeficiency mice, then found that in a total of 21 T-cell lines, 60% were CD4^+^ and 13% were CD8 + .

These results showed that the generation of CD8^+^ T-cell lines and clones was more difficult than that of CD4^+^ T cells in MG/EAMG. However, no abovementioned reports described the reason; thus, we suggest that the method of EAMG induction or cultural condition of T-cell clone selection from patients with MG or EAMG might favor CD4^+^ T cells, which is similar to our findings in EAE and EAU [31, 32, 34, 55].

## Major TCR V gene usage of CD8^+^ T cells in MG

In the thymus, human thymocytes rearrange V and J segments to create a functional heterodimeric α/β TCR, which is expressed as a disulfide-linked heterodimer on the mature T cell to be recognized as antigen peptides by self-MHC. TCR V gene usage is critical for the pathogenesis of autoimmune diseases, including MG. The autoreactive CD8^+^ cells from patients with MG might have specific Vα and Vβ gene usage [56].

Compared with CD8^+^ cells from HC, several reports showed different patterns in the Vβ gene usage of CD8^+^ cells from patients with MG, such as (1) Vαl2.1, and Vβ6.7, 8, 12; (2) Vβ1, 13.2, 17, and 20 [56]; (3) Vβ10, 13, and 17; (4) vβ3 and Vβ19; (5) Vα2.3, 12.1, and β2, 3, 5.1, 5.2, 5.3, 6.7, 8, 13, and 17. For different subtypes of MG, remarkable CD8^+^ TCR Vβ-subset expansion was found in 64% and 72% of late-onset MG or thymoma-associated MG (vs. 16% with early-onset MG) [57].

The real pattern of the Vβ gene usage of CD8^+^ cells from patients with MG is difficult to determine because results were obtained from different groups, which might have used different standards of testing. Moreover, due to the lack of reports in the past and the last decade, this topic must be studied further.

## Cytokine and chemokine secretion of CD8^+^ T cells in MG/EAMG

### Patients with MG

In patients with MG with MuSK, tacrolimus inhibited CD8^+^ T-cell proliferation and interferon (IFN)-γ and IL-2 production; however, tacrolimus inhibition was lower in CD4^+^ T cells [58, 59]. Nevertheless, these results had two shortcomings: [1] CD8^+^ T cells were not highly purified using fluorescence-activated cell sorting or magnetic separation enrichment but CD3^+^CD8^+^ were only separated from whole PBMCs using flow cytometry. [2] These PBMCs were stimulated with either αCD3 and αCD28 or phorbol 12-myristate 13-acetate and ionomycin in the presence of brefeldin A but not with autoantigens such as AchR. As we have previously performed in EAE and EAU, these T cells should be dominated by non-Ag-specific T cells, which are not able to induce diseases [31, 34, 55, 60].

Intracellular staining showed that the ratio of Th2 decreased significantly, and Th1 and Th17 were significantly increased in patients with MG [24]. IL-17-producing CD4^+^ T cells (Th17) increased significantly with disease severity in patients with MG [36, 59]. Similarly, CD8^+^ T-cell subsets producing IL-17 and IL-17/IFN-γ increased in patients with MG, whereas the increased cell levels were inhibited by the retinoic acid receptor-related-orphan-receptor-C (RORγ) inhibitor. These findings provide a rationale for the exploration of targeted Th17 therapies, including ROR-γ inhibitors, to treat patients with MG [61].

### EAMG

Compared with female C57BL/6 J wild-type (*wt*) mice, the clinical EAMG score, AChR-specific T and B cell responses, and AChR-reactive IFN-γ and IL-4-expressing cells in lymphoid organs were inhibited in CD4^−^8^−^, CD4^−/−^, and CD8^−/−^ mice. The results suggest that both CD4^+^ and CD8^+^ T cells are essential for EAMG development. Another report showed that there was earlier development of EMG and more serum anti-AChR Abs in IL-10 transgenic C57BL/6 mice and that CD8^+^-depleted splenocytes secreted markedly more IL-10 and lesser IFN-γ in vitro when stimulated with AChR from AChR-immunized transgenic mice [62].

## T-cell marker expression of CD8^+^ T cells in MG

### CD45RA

CD45, the tyrosine phosphatase receptor type C (PTPRC) protein, is an essential molecule involved in thymocyte maturation. CD45RA^+^ usually stands for the “naive” subset of CD4^+^ T cells, and CD45RO^+^ is major for “memory” T cells. Compared with HC, CD8^+^ but not CD4^+^ subsets among CD45RA^+^ T cells increased in the blood of patients with thymoma. Interestingly, after thymoma resection, the CD8^+^ but not CD4^+^ subset of CD45RA^+^ T cells in the blood decreased [63]. Furthermore, the ratio of CD45RO^+^ to CD45RA^+^ T cells was lower in CD8^+^ T cells of patients with early onset MG than in those of patients with late-onset MG and thymoma [64]. CD8^+^CD45RA^+^ lymphocytes in the muscle of patients with MG may signify an underlying thymoma and should not be misdiagnosed as polymyositis (PM) because all T lymphocytes in PM cases were CD45RA^−^ [65]. Furthermore, in late-onset MG, approximately 25% of the expanded cells initially exhibited a naïve CD62L^+hi^/CD45RA^+^ recent thymic emigrant-like phenotype. These expansions were significantly associated with IgG antibodies against cytomegalovirus, IL-12, and IFN-α2. The CD8^+^ TCRVβ expansion was stable over five years; however, recent thymic emigrant markers declined [66]. A significantly lower number of naïve helper T cells (CD4^+^CD45RA^+^) with an increased proportion of memory helper T cells (CD4^+^CD45RO^+^) and a significantly lower number of naïve cytotoxic T cells (CD8^+^CD27^+^CD28^+^) were observed in patients that had undergone thymectomy. Their findings indicated the premature aging of the immune system after Tx in juvenile MG; however, the associated clinical consequences could not be verified [51].

### Costimulatory molecules

CD8^+^ cells of patients with MG expressed lower CD28 and higher CD80 and CD86. Furthermore, there were lower percentages of CD8^+^CD86^+^ Tcells in patients with an early onset of MG (< 40 years) compared with those with a late onset of MG (> 40 years). These data indicate that the CD28/CD80–CD86 costimulatory pathway is involved in MG [67]. The percentages of CD4^+^ and CD8^+^ T lymphocytes expressing the CD28 antigen were significantly lower in patients that had undergone thymectomy than in healthy subjects [68].

### Chemokine receptors

Before therapy, patients with MG showed a reduction in the frequency of chemokine receptor 3 (CXCR3)^+^ CD4^+^ T cells, especially in the thymoma group; however, CXCR3^+^ CD8^+^ T cells remained normal. Conversely, after therapy, in the hyperplasia group, the expression of chemokine receptor 1 (CCR1) in CD4^+^ and CD8^+^ cells significantly increased and then returned to control levels [69]. CD4^+^ CXCR5^+^ T cells were mainly found in patients with MG; however, CD8^+^ T cells were predominant in HC [70]. In patients with MG, B and dendritic cells showed significantly higher levels of glycolysis and glycolytic capacity than CD8^+^ T cells, CD4^+^ T cells, and their subsets [24].

### Others

Higher expression levels of estrogen receptors were observed in CD4^+^ helper T cells than in CD8^+^ cells obtained from the PBMCs of patients with MG [71]. The IFN-γ-inducible protein 10 (CXCL10) receptor and CXCR3 expression in EAMG rats increased markedly only in CD4^+^ and not in CD8^+^ T cells or CD19^+^ B cells [72]. Fas expression in peripheral CD8^+^ T cells was higher in patients with MG with a normal thymus than in patients with MG with thymoma and controls. Moreover, Fas expression in CD8^+^ cells was significantly higher in patients with MG treated with corticosteroids than in controls [73].

Effector CD4^+^ T cells expressing CD57^+^, CD57^+^ killer cell lectin-like receptor G1^+^, CD57^+^TIGIT^+^, CTLA-4, EOMES, FOXP3, and PD-1 increased after eculizumab treatment. However, HLA-DR expression on CD8^+^ T cells decreased or remained at low levels, and a mild-to-moderate-level increase in PD-1 expression was observed in CD8^+^ T cells after eculizumab treatment. Furthermore, C5aR was expressed on CD4^+^ and CD8^+^ T cells at low levels before and after eculizumab treatment. CD8^+^, CD4^+^CD8^+^, and CD4^+^CD8^+^CXCR5^+^ T-cell populations decreased after eculizumab treatment. CD8^+^ T cells expressing CD57^+^TIGIT + CD27^low^ CD28^low^ increased after eculizumab treatment [13]. In patients with MG, the expression of PI3KCA, AKT-1, mTORC1, HIF-1α, GLUT1, HK, PFK, and PK increased significantly in Th1, Th17, and CD4^+^ CD25^−^ cells, and the expression of AKT-1, HIF-1α, GLUT1, PK, and CPT1A increased significantly in CD4^+^ CD25^+^ Tregs. However, there were no significant changes in the above signaling pathways in any subset of CD8^+^ T cells [24].

## Regulatory CD8^+^ T cells in MG

Tregs play a critical role in regulating immune tolerance and preventing autoimmunity, and CD4^+^ Foxp3^+^ Tregs have been well-studied [74, 75]. Recent studies on CD8^+^ Tregs have shown good improvements [74]. Some CD8 + T cell subsets have been defined as CD8^+^ Tregs from different reports in different experimental systems [74]. For example, CD8^+^ Foxp3^+^ Tregs are involved in tissue transplantation and alloantigen-induced immune responses [76–81]. CD8^+^ CD103^+^ cells were generated from naïve CD8^+^ T cells cultured with TGF-β in vitro and showed inhibition in vivo, as well as in an aggressive tumor model, and expressed Foxp3 [82–84]; CD8^+^ CD28^−^ T cells were found in age-dependent accumulation and chronic antigen exposure [85–87]. CD8^+^ CD122^+^ CD49d^+^ cells express both PD-1 and IL-10 and inhibit alloantigen-induced transplant rejection [88–91]. CD8^+^ CD122^hi^ Ly49^+^ cells were discovered in young naïve mice [92, 93], EAE models [92, 94, 95], colitis [96, 97], hepatitis [98], arthritis [99], diabetes [100, 101], viral infection [102], tumor immunity [103], atherogenesis [104], and organ transplantation [105].

CD4^+^CD25^high^Foxp3^+^ Tregs decreased in patients with MG; however, CD8^+^CD28^−^ and CD8^+^CD122^+^ Tregs do not change significantly [106]. Tacrolimus suppressed CD4^+^ regulatory and helper T cells in MG, such as Tregs (CD4^+^CD25^+^FOXP3^+^), peripheral blood Tfh (Tfh-like cells: CD4^+^CXCR5^+^), and follicular Tregs (CD4^+^CXCR5^+^FOXP3^+^) [59]. The percentage of CD4^+^CD25^+^CD127^−^ Tregs in the peripheral blood of patients with generalized MG was significantly lower than that in patients with OMG and HCs [43]. The populations of CD8^+^CD28^−^ and CD8^+^CD122^+^ Tregs did not differ between patients with MG and healthy controls; patients with MG exhibited a decrease in CD4^+^CD25^high^Foxp3^+^ Tregs and an increase in CD19^+^BAFF-R^+^ B cells, revealing that patients with MG should display the dysfunction of T-cell balance and the activation of B-cell maturation [106]. The dual APL unregulated Foxp3 expression in CD8^+^CD28^−^ cells and the secretion of TGF-β and IL-10 are independent of CD8 cells. In TAChR-immunized CD8^−^/^−^ knockout mice, the inhibitory effect of dual APL failed, the expression of caspase 3 and 8 was unregulated, the expression of Bcl-xL was downregulated, and CD4^+^CD25^+^ Tregs were induced by dual APL. This suggests that CD8^+^CD28^−^ regulatory cells play a partial role in inhibiting EAMG via dual APL [107].

## CD20-expressing T cells (or CD20^+^ T cells) in autoimmune diseases

Anti-CD20 mAbs is a potential therapy for both antibody-mediated and T cell-mediated autoimmune diseases [108], such as MS [109–112], rheumatoid arthritis (RA) [113–115], systemic lupus erythematosus [115–117], antineutrophil cytoplasmic antibody-associated vasculitis [117, 118], polymyositis/dermatomyositis [119, 120], primary Sjögren's syndrome [121, 122], idiopathic autoimmune thrombocytopenia and neutropenia [123], and MG [119, 124, 125]. Two mechanisms might explain the effect of rituximab on T cells: one is the indirect effect on B cell depletion that could influence T cell function, while the other, which might be more important, is the direct depletion of CD20 + T cells [126].

Typically, CD20 is expressed by mature B cells [127, 128]. However, there are some reports of CD20 being expressed by some T cells, which are called CD20-expressing T cells (CD3^+^ CD20^+^ T cells). CD20-expressing T cells were first reported in T cell lymphoma [129], RA [130] and animal-model experimental arthritis (EA) [131], MS [126, 132–136], EAE [136], and neuromyelitis optical spectrum disorder (NMOSD) [132], as well as in healthy individuals (3–5% of all CD3^+^ cells) [137]. Furthermore, CD3^+^ B cells have been reported [138].

### MS and EAE

In MS patients, CD20-expressing T cells have specific characteristics [132, 134, 135, 139]: located at the thymus, bone marrow, and secondary lymphatic organs, as well as CSF, even without inflammation [132, 135]; expressed CD8-related cytotoxic program (granzyme-B, Perforin, Runx3, IRF4, CD28, CD56, CD57, CD94, CD150, CD215) [139]; enriched in CD8 + and CD45RO + memory cells and in CCR7- cells [132]; high secretion of IL-4–, IL-17–, IFN-γ, and TNF-α [132]; reduced by rituximab [126], fingolimod [132], alemtuzumab [132], dimethyl fumarate [132] [135], and ocrelizumab [134], but increased by natalizumab [132]. Another study provided more evidence of CD20-expressing T cells in MS patients. At the pretreatment stage, higher frequencies of CD20dimCD8 + T cells were related to a higher concentration of myelin basic protein in CSF, higher gadolinium-lesion counts, higher T2-weighted lesion volume, and lower normal appearing white matter and thalamus volume in MRI; this might predict outcomes of anti-CD20 treatment [135]. Depletion of CD20dimCD8 + T cells could improve outcomes of anti-CD20 treatment. Furthermore, anti-CD20 treatment was not able to reconstitute CD20dimCD8 + T cells [140].

CD20 + T cells were also found in active EAE mice, and the adoptive transfer of CD20 + T cells into EAE mice enhanced the disease without the assistance of B cells [136].

### RA and EA or murine collagen-induced arthritis (CIA)

In peripheral blood T cells, frequencies of CD20-expressing T cells were 0.1–6.8% in healthy individuals and 0.4–2.6% in RA patients [130]. These CD20 + T cells co-expressed CD8 (45%) and CD4 (55%), as well as differentiation/activation associated markers (CD29, CD38, CD45RO, CD49a, CD56, CD69, CD154, CD161, and CD166), but did not express other B cell markers (CD19, CD21, CD24, or soluble IgM). These CD20 + T cells secreted IFN-γ, IL-1, IL-2, IL-4, IL-8, IL-10, IL-17, MCP-1, TGF-β, and TNF-α; increased calcium flux; and easily entered apoptosis under stimulation, compared to CD20- T cells [130].

## CXCR5^+^ CD8^+^ T cells in autoimmune diseases

Recently, a novel subset of CD8 T cells, CXCR5 + CD8^+^ T cells, were identified [141–143]. CXCR5 + CD8^+^ T cells have been found to infiltrate the B cell follicle in response to several diseases, including viral infection (simian immunodeficiency virus (SIV) or human immunodeficiency virus (HIV) [144–170], lymphocytic choriomeningitis virus (LCMV) [149, 171, 172], hepatitis B virus (HBV) [173–180], polycythemia-inducing FV [180], FluA [181, 182], HSV [183], DENV2 [184, 185], and SARS CoV-2 [186–190], EBV [191]); cancer (colorectal cancer [192–194], NCLC [195], hepatocellular carcinoma [175, 196], hematologic malignancies [197], pancreatic tumors [198], gastric cancer [199], breast cancer [200], thyroid cancer [201], melanoma [202], and lymphoma [203]); bacterial infection (*Escherichia coli*, *Acinetobacter baumannii*, *Klebsiella pneumonia*, *Pseudomonas aeruginosa*, and *Staphylococcus aureus* [204]); parasitic infection (*Leishmania mexicana* [205]); immunodeficiency disease (CVID [206]); autoimmune diseases [4, 131, 207–210] (rheumatoid arthritis (RA) [131, 207] [211], primary Sjögren syndrome (pSS) [208] [212], and multiple sclerosis (MS) [210, 213]).

CXCR5^+^ CD8 T cells have special phenotypes: for example, 1) cell markers including CD11a, CD11b, CD20, CD25, CD27, CD28, CD38, CD39, CD40, CD43, CD44hi, CD45RA, CD45RO, CD57, CD62L, CD69, CD83, CD94, CD95, CD101, CD103, CXCR5,CD107, CD127, CD137, CD161, CD200, CD244, HLA-DR, 41BBL, Slamf6, and TCRVa7.2 [13, 131, 145, 146, 149, 151–155, 157–160, 163–165, 167, 168, 171, 174, 176–178, 180–182, 184–190, 193–195, 197, 199, 201, 204, 205, 207, 211, 212, 214]; 2) cytokines, chemokines and receptors (CSF-1, IL-2, IL-4, IL-6, IL-7, IL-7R, IL-10, IL-17, IL-18Ra, IL-21, IL-21R, IL-22, IL-23, IL-27, IL-27R, IL-35, IFN-I, IFNAR1, IFN-γ, TGF-β, TNF-α, IL-4R (CD124), CCR2, CCR4, CCR5, CCR6, CCR7, CCR9, CCL5, CXCL1, CXCL5, CXCL10, CXCL12 (SDF-1α), CXCL13, CXCR3, CXCR4, CXCR5, CX3CR1, MIP-1β) [144–146, 149, 151, 153, 154, 157, 159–162, 165, 167, 168, 170, 172–174, 176, 177, 179, 181–186, 188, 190, 191, 193–197, 199, 200, 205, 206, 209, 211, 212]; 3) transcription factors (Bcl-6, CDCA7, CTLA-4, CULT1, E2A, pERK1/2, granulysin, granzyme B, Helios, ICOS, Id2, ISGs, interferon pathway associated molecules (MX1, MX2, GBP1, and ISG15), Ki-67, KLRG1, LAG-3, MAMU-DRA, MEF2C, NFATC1, NFATC2, PD-1, PD-L1, PD-L2, perforin, PRDM1, RANTES, SOX4, SPRY2, STAT2, STAT6, TCF4, TCF24, and Tim-3) [145, 146, 148, 149, 151–158, 162–166, 168, 172, 174, 175, 178, 211].

## Conclusion

In this review, we focused on the updated information on CD8^+^ T cells in MG/EAMG, as well as on the relevant evidence (peripheral, in situ, CSF, and under different conditions), in vitro culture, TCR usage, cytokine and chemokine expression, cell marker expression, Tregs, and Tc17. However, the mechanism underlying the regulatory role of CD8^+^ T cells in MG/EAMG remains unclear. In addition, the following questions should be addressed in future studies: (1) Why are fewer studies available on CD8^+^ T cells in MG than those on CD4^+^ T cells in MG?; (2) Is the classical T-cell culture system favorable to CD4^+^ T cells compared to CD8^+^ T cells, and is it similar to our experience with studies on CD8^+^ T cells in EAE and EAU [31, 32, 34, 55]?; (3) What is the real pattern of the Vβ gene usage of autoantigen-specific CD8^+^ cells from patients with MG?; (4) As CD8^+^ T cells were not highly purified and were not stimulated by autoantigens, what are the real images of the physiology and function of autoantigen-specific CD8^+^ cells from MG/EAMG available?; (5) Which subset of autoantigen-specific CD8^+^ cells (Tc1, Tc17, IL-17^+^IFN-γ^+^CD8^+^ T cells, or IL-17^+^IFN-γ^+^TNF-α^+^CD8^+^ T cells) plays the most critical role in the pathogenesis of MG/EAMG? Did they like the similar studies that have been performed on EAE, diabetes, and systemic lupus erythematous disease [32]; (6) Which subsets of autoantigen-specific CD8^+^ Tregs play a regulatory role in MG/EAMG among CD8^+^CD28, CD8^+^CD122^+^ [106], CD8^+^ CD122^+^ CD49d^+^ cells [88–91], or CD8^+^ CD122^hi^ Ly49^+^ cells [92–105], which have been well studied in other diseases. Hence, it is necessary to perform further studies on CD8^+^ T cells in MG/EAMG, especially on autoantigen-specific CD8^+^ Tregs.

In additional, there are many reports of CD20-expressing T cells (or CD20 + T cells) and CXCR5^+^ CD8 T cells on autoimmune diseases, especially on MS and RA. Unfortunately, up to now, there has been no report on these T cells on MG, which may be a good direction for future studies on MG.

## Data Availability

All data generated or analyzed during this study are included in this published article. The data supporting this article are listed within the article. For additional information, please contact the corresponding author.

## Abbreviations

Ab: Autoantibody
AChR: Muscle nicotinic acetylcholine receptor
Ag: Antigen
CSF: Cerebrospinal fluid
CTL: Cytotoxic T-lymphocytes
EAE: Experimental autoimmune encephalomyelitis
EAMG: Experimental autoimmune myasthenia gravis
EAU: Experimental autoimmune uveitis
HC: Healthy control
IFN: Interferon
IL: Interleukin
MG: Myasthenia gravis
MHC: Major histocompatibility complex
MuSK: Muscle-specific tyrosine kinase
OMG: Ocular MG
PBMCs: Peripheral blood mononuclear cells
RORγ: Retinoic acid receptor-related-orphan-receptor-C
TCR: T-cell receptors
Tfh: Follicular Th
Th: T helper
Th17: IL-17-producing CD4^+^ T cells
Tregs: Regulatory T cells
Tx: Thymectomy
